# Marine Oil Pollution Monitoring Based on a Morphological Attention U-Net Using SAR Images

**DOI:** 10.3390/s24206768

**Published:** 2024-10-21

**Authors:** Lena Chang, Yi-Ting Chen, Ching-Min Cheng, Yang-Lang Chang, Shang-Chih Ma

**Affiliations:** 1 Department of Communications, Navigation and Control Engineering, National Taiwan Ocean University, Keelung 202301, Taiwan; 2 The Intelligent Maritime Research Center (IMRC), National Taiwan Ocean University, Keelung 202301, Taiwan; 3 Department of Electrical Engineering, National Taiwan Ocean University, Keelung 202301, Taiwan; 20953003@mail.ntou.edu.tw; 4 Department of Electrical Engineering, Chang Gung University, Taoyuan City 33302, Taiwan; cmc0729@cht.com.tw; 5 Department of Electrical Engineering, National Taipei University of Technology, Taipei 10608, Taiwan; ylchang@mail.ntut.edu.tw (Y.-L.C.); scma@ntut.edu.tw (S.-C.M.)

**Keywords:** oil spills, U-Net model, synthetic aperture radar (SAR), convolutional block attention module (CBAM), label smoothing

## Abstract

This study proposed an improved full-scale aggregated MobileUNet (FA-MobileUNet) model to achieve more complete detection results of oil spill areas using synthetic aperture radar (SAR) images. The convolutional block attention module (CBAM) in the FA-MobileUNet was modified based on morphological concepts. By introducing the morphological attention module (MAM), the improved FA-MobileUNet model can reduce the fragments and holes in the detection results, providing complete oil spill areas which were more suitable for describing the location and scope of oil pollution incidents. In addition, to overcome the inherent category imbalance of the dataset, label smoothing was applied in model training to reduce the model’s overconfidence in majority class samples while improving the model’s generalization ability. The detection performance of the improved FA-MobileUNet model reached an mIoU (mean intersection over union) of 84.55%, which was 17.15% higher than that of the original U-Net model. The effectiveness of the proposed model was then verified using the oil pollution incidents that significantly impacted Taiwan’s marine environment. Experimental results showed that the extent of the detected oil spill was consistent with the oil pollution area recorded in the incident reports.

## 1. Introduction

According to the records of the International Marine Organization (IMO), more than 90% of global trade and transportation rely on maritime shipping, as it is the most cost-effective and efficient method for international trade [1]. The rapid development of maritime transportation has had a significant impact on marine environmental pollution. Oil spills are one of the primary causes of marine environmental pollution. The sources of marine oil pollution include offshore platforms, ship accidents, illegal discharges from ships, leaks from submarine oil pipelines and port operations, etc. Among them, industrial facilities emit the most oil pollution, while oil pollution caused by ship accidents or illegal discharges occurs more frequently [2]. Oil pollution not only harms the environment and marine life but also requires substantial manpower and resources for cleanup.

In the past, the inspection of oil spills on the sea surface by aircraft and ships was relatively flexible. However, due to the small observation range of these navigation vehicles, it was time-consuming to patrol the entire sea area. In addition, oil pollution inspection and monitoring by aircraft and ships were mainly based on visible light or infrared sensors, which could not effectively detect oil spills at night or in severe weather conditions. In recent years, spaceborne synthetic aperture radar (SAR) imagery has played a significant role in marine environment monitoring due to its high resolution and wide coverage, even under adverse weather conditions. Oil spills on the sea surface inhibit capillary waves and reduce radar backscatter. Consequently, oil spill areas appear as dark regions in SAR images [3,4]. Therefore, it is possible to achieve 24-h all-weather marine oil spill observation through SAR images.

In the past few years, many researchers have engaged in oil spill detection using SAR data. Migliaccio et al. [5] combined the constant false alarm rate (CFAR) and target decomposition (TD) theorem to classify oil spills using SIR-C/X SAR data. Nunziata et al. [6] utilized the co-polarized phase difference (CPD) model to distinguish the oil spills and biogenic slicks from SIR-C/X SAR data. Chang et al. [7] proposed a region-based SAR oil spill detection method. This method combined the image segmentation and the generalized likelihood ratio test method for oil spill detection. Moreover, Shirvany et al. [8] conducted the experiments of oil spill detection by using the degree of polarization (DoP) on RADARSAT-2 and NASA/JPL UAVSAR data. Xu et al. [9] compared the oil spill detection performance using several classification methods, including a support vector machine (SVM), tree-based ensemble classifiers, generalized additive model (GAM) and penalized linear discriminant analysis (PLDA) using RADARSAT-1 imagery.

Recently, deep learning has been widely applied to SAR image segmentation and classification tasks and has achieved superior performance. Many studies have adopted automatic approaches, such as convolutional neural networks (CNNs) or deep convolutional neural networks (DNNs), to extract features for SAR oil spill detection, replacing the handcrafted features used in traditional methods [10,11,12,13,14,15,16,17]. Among them, the fully connected network (FCN) architecture has demonstrated its ability to perform accurate image segmentation in oil spill detection, especially when used in conjunction with the U-Net model. In recent years, many studies have also proposed SAR oil spill detection methods based on the U-Net model. Ronci et al. [18] introduced an innovative solution based on image-to-image translation using a U-Net model trained with an adversarial loss function. The proposed method was tested using Radarsat-2 and Sentinel-1 SAR data from the Mediterranean Sea, the Atlantic Ocean, and the North Sea, effectively improving oil spill detection results. Chen et al. [19] proposed an oil spill detection method based on the U-Net model and conducted experiments using Sentinel-1 polarimetric SAR (PolSAR) satellite imagery. In addition to considering the polarization information of the PolSAR dataset and wind speed data, the model also incorporated attention gates to enhance feature extraction capabilities. Li et al. [20] developed a novel self-evolving algorithm for automatic oil spill detection, which was composed of three interconnected modules. The algorithm automatically detected oil spills and generated additional training data through an adaptive thresholding method to enhance the training capacity of the deep learning model. Li et al. [21] improved the U-Net model by incorporating additional modules to improve feature extraction capabilities, resulting in more accurate oil spill segmentation. By fusing feature maps from different levels in the U-Net decoder, the network could learn rich global and local contextual information, which effectively enhanced its ability to recognize complex textures and ambiguous boundaries, thereby improving the segmentation accuracy of SAR images. Rubicel et al. [22] combined the MLP and U-Net models for oil spill detection and adjusted the number of convolutional layers, filters, hidden layers, and neurons at each layer. In this way, the oil spill detection model reduced the false positives caused by lookalikes, and effectively improved the overall detection performance.

Chen et al. [23] proposed the full-scale aggregated MobileUNet (FA-MobileUNet) model for oil spill detection. FA-MobileUNet incorporated the convolutional block attention module (CBAM), the atrous spatial pyramid pooling (ASPP) module, and the full-scale aggregation (FA) module into U-Net to effectively extract the semantic features of each category. The FA-MobileUNet model can effectively distinguish lookalikes that are often confused with oil spills, significantly improving the efficiency of oil spill detection. However, segmentation results of SAR images often suffer from fragmented regions and incomplete shapes, which may be caused by inherent non-stationary and non-uniform sea clutter. Fragmented oil spill detection results are less favorable in describing the location and extent of the oil pollution event. Thus, this study further improved the performance of FA-MobileUNet in oil spill detection, especially in reducing fragmented detection results. The concept of morphology was introduced to improve the spatial feature extraction of the model, which can produce more complete feature maps during the training process, thereby resulting in a more comprehensive oil spill detection area. The experiments on oil spill incidents verified the effectiveness of this improved FA-MobileUNet in monitoring marine oil pollution.

The rest of this paper is organized as follows. Section 2 describes the oil spill dataset used in this study and introduces the proposed oil spill detection method. The experimental results are shown in Section 3. Some discussions are presented in Section 4. Finally, the study is concluded in Section 5.

## 2. Materials and Methods

### 2.1. Oil Spill Dataset

Due to the lack of a common oil spill dataset, the performance of previous studies [10,11,12,16,17] cannot be effectively compared and analyzed. Moreover, the extent of oil spill areas in the ocean is relatively small, making it difficult to obtain a large amount of data to train deep learning networks. Krestenitis et al. [24] created a common dataset for oil spill detection using Sentinel-1 SAR images from the Copernicus Open Access Hub [25] by the European Space Agency (ESA), and this is available on their public website [26]. The data from MKLab were collected from oil spill incidents recorded by EMSA’s CleanSeaNet system. Sentinel-1 images corresponding to the timestamp and geolocation of the oil spill incidents were downloaded for identification. The data collection period for the original MKLab dataset ranged from September 2015 to October 2017. Chen et al. [23] augmented the MKLab dataset by searching for marine oil spill events from 2015 to 2022 and obtaining Sentinel-1 images from the ESA website based on the location and time of the events. In the study, the oil spill detection model was trained using the extended MKLab dataset [23].

**Figure 1:**
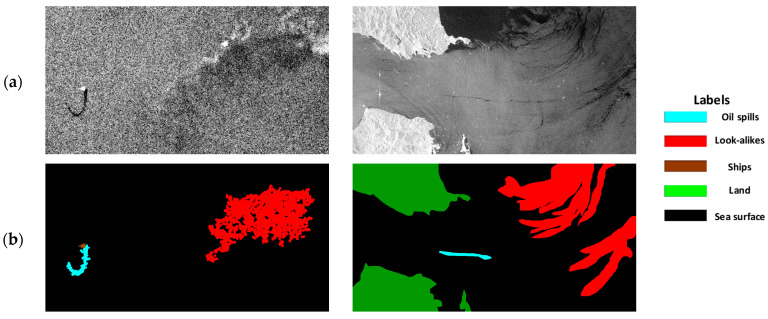
Oil spill images from the dataset. Cyan, red, brown, green, and black correspond to oil spills, lookalikes, ships, land, and sea surface, respectively. (**a**) Original SAR image; (**b**) ground truth data.

The extended oil spill dataset contained a total of 1239 images with a size of 1250 × 650 pixels, including 1129 training images and 110 testing images. This dataset was composed of Sentinel-1 VV polarization and C-band images with a pixel spacing of 10 m × 10 m. The dataset included five categories: oil spills, lookalikes, ships, sea surface, and land. Each category was annotated with different RGB colors: oil spills in cyan, lookalikes in red, ships in brown, sea surface in black, and land in green, as shown in Figure 1. Moreover, one-dimensional annotation data were provided, assigning each category an integer value between 0 and 4.

### 2.2. Improved Oil Spill Detection Model

**Figure 2:**
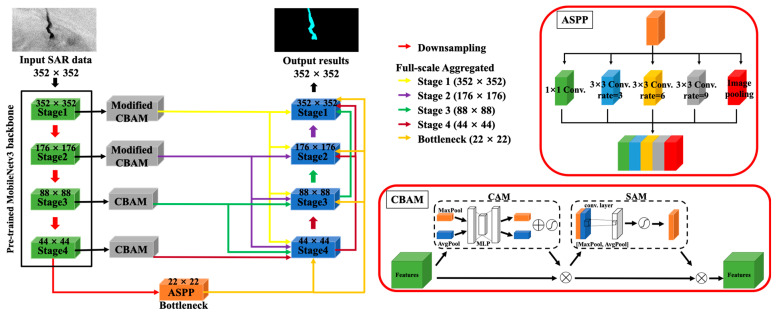
The structure of the improved FA-MobileUNet model.

Semantic segmentation networks have been widely applied to SAR image oil spill segmentation in recent years, achieving excellent detection performance. The U-Net model [27,28], an encoder–decoder architecture, can be trained with a small amount of data to yield fast and accurate segmentation results. In addition, a study [23] further modified the original U-Net model, effectively improving the detection performance for oil spills and lookalikes. This modified model enhanced feature extraction for targets at different scales and provided information of various categories for model learning. Moreover, the model can effectively distinguish lookalikes that are often confused with oil spills, significantly improving the efficiency of oil spill detection. However, due to the irregular roughness of the sea surface caused by wave fluctuations and wind conditions, the SAR backscattering value in the oil spill area changes greatly, resulting in fragmented detection results in the oil spill area. For example, there were some small holes within the detected oil spill or lookalike areas, as shown in Figure 1b. Complete oil spill detection range facilitates shape description and area reporting of oil spills in marine environment monitoring. Therefore, to monitor marine oil spill pollution more effectively, the concept of morphology was introduced into FA-MobileUNet to enhance the spatial information extraction capability of the model and obtain more complete oil spill segmentation results. The architecture of the improved FA-MobileUNet model was shown in Figure 2.

#### 2.2.1. FA-MobileUNet Model

The U-Net model can provide high-resolution segmentation results due to its encoder–decoder architecture and the skip connection approach. The encoder effectively captures the contextual information of the input data, while the decoder utilizes this extracted information to produce accurate segmentation results. The FA-MobileUNet model enhanced feature extraction capabilities by replacing the encoder’s backbone network with MobileNetv3 [29]. MobileNetv3 is a convolutional neural network (CNN) architecture designed for mobile and edge devices, focusing on achieving a balance between low latency and high accuracy. MobileNetv3 relies heavily on depthwise separable convolutions to reduce computational complexity. In addition, inverted residual blocks and squeeze-and-excitation (SE) modules [30] are utilized to enhance feature extraction capabilities without increasing computational burden.

Next, the attention mechanisms in deep learning models were developed to enhance the model’s ability to focus on relevant parts of the input data, thereby improving performance and interpretability. Attention mechanisms allow models to dynamically prioritize different parts of the input data when producing elements of the output. This approach helps the model focus on the most relevant contextual information in the input data and extract meaningful features. Therefore, the FA-MobileUNet model adopted the convolutional block attention module (CBAM) [31], which consisted of the channel attention module (CAM) and the spatial attention module (SAM), to enhance the feature representation of the feature maps output by the encoder.

In addition, atrous spatial pyramid pooling (ASPP) is a key feature extraction module in deep learning, especially in the context of semantic segmentation tasks [32]. Its main purpose is to capture multi-scale contextual information and improve the ability of CNNs to segment images at different scales. Atrous convolutions enable ASPP to increase the receptive field without reducing the spatial resolution of feature maps. Therefore, the bottleneck connecting the encoder and decoder in the FA-MobileUNet model incorporated the ASPP module to enhance multi-scale feature extraction.

**Figure 3:**
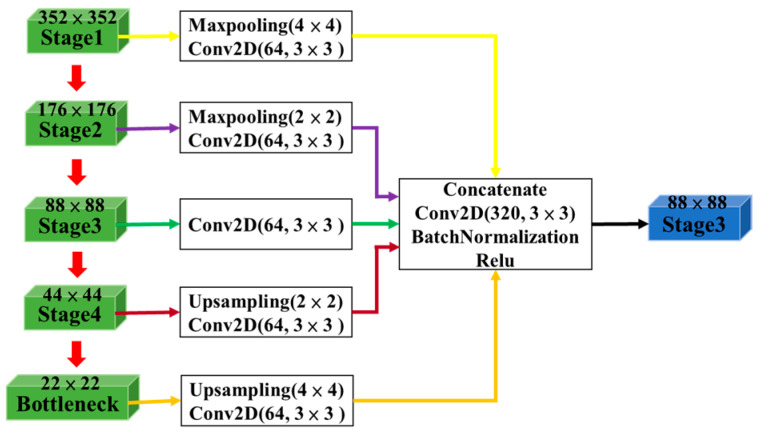
The structure of the FA module of stage 3.

However, as the U-Net model passes through multiple convolutional layers and downsampling, meaningful features gradually disappear and the spatial resolution decreases. Therefore, to effectively utilize low-level spatial information and high-level semantic features, the full-scale aggregated (FA) module concatenated the features at different scales of the encoder block and aggregated them with the feature maps of the decoder block, as shown in Figure 3. The FA approach allowed the model to effectively train targets at different scales, thereby improving oil spill detection performance. Consequently, the model can effectively utilize feature maps of different scales for training, resulting in accurate detection results.

#### 2.2.2. Modified CBAM

The segmentation results of SAR maritime images often suffer from numerous imperfections, such as fragmented regions and incomplete shapes. These deficiencies may arise from inherent non-stationary and non-uniform sea clutter. As shown in the experiments in Section 3, there are some defects in the segmentation results, including incomplete oil spill regions or some small holes within the oil spill and lookalike areas.

Morphological image processing can focus on the shape or structure of features in an image through nonlinear operations. For example, the closing operation can provide continuous and smooth region boundaries, and the hole filling operation can be used to fill small holes inside an object while retaining the shape and size of the original object. Therefore, this effective morphological image processing can provide a feasible solution to the imperfections of SAR image segmentation. Previous studies [33,34,35] used the morphological dilation and erosion operations to replace the convolutional layers to verify their effectiveness in feature extraction and noise removal. To improve the performance of the FA-MobileUNet model, this study referred to the concept of morphology to enhance the feature extraction of spatial information. Morphological operations slide structural elements across the image, similar to convolution operations using kernel functions, highlighting spatial features in the output. Erosion2D (⊖) and Dilation2D (⊕) operations on an input image *I* are defined as follows:

(1) $$\left(I⊖K_{e}\right)\left(x,y\right)=\underset{m\in S_{1},n\in S_{2}}{\min}\left(I\left(x+m,y+n\right)-K_{e}\left(m,n\right)\right)$$

(2) $$\left(I⊕K_{d}\right)\left(x,y\right)=\underset{m\in S_{1},n\in S_{2}}{\max}\left(I\left(x-m,y-n\right)+K_{d}\left(m,n\right)\right)$$

where $K_{e}$ and $K_{d}$ are the structuring elements of erosion and dilation operations of size a×b, $K_{e},K_{d}\in R^{a\times b}$, $S_{1}=\{1,2\ldots ,a\}$, and $S_{2}=\{1,2\ldots ,b\}$. In addition, the closing operation involves a dilation operation followed by an erosion operation.

**Figure 4:**
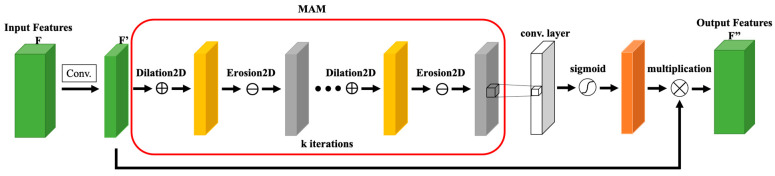
The structure of the MAM.

This study improved the FA-MobileUNet model by replacing the spatial attention module (SAM) in the original CBAM with the morphological attention module (MAM) to further enhance attention to spatial features. The closing operations was applied in MAM, which replaced the convolutional layers in SAM. The structure of MAM was shown in Figure 4. The initial kernel size of the morphological operations was set to 3 × 3, allowing for adjustment during training. As shown in Figure 4, the input feature map *F* was first processed by a 1 × 1 convolution layer $W_{1}$, and then the morphological closing operations were performed through a series of Dilation2D and Erosion2D layers. The output of MAM can be expressed as follows:

(3) $$M_{M}(F)=F\times W_{1}\times K_{c}\times \ldots \times K_{c}$$

where $F\in R^{H\times W\times C}$, $W_{1}\in R^{1\times 1\times C}$, and $M_{M}(F)\in R^{H\times W\times 1}$. $K_{c}$ is the morphological closing operation. Finally, the morphological feature maps were then processed through a 1 × 1 convolution employing the Sigmoid activation function. However, the closing operation may lose some details while maintaining spatial shape and structural characteristics. Paying attention to these two features simultaneously, and based on the experimental results in Section 3, it was proposed to enhance the low-level spatial features extracted from Stages 1 and 2 of the encoder through the modified CBAM, while processing the high-level semantic features extracted from Stages 3 and 4 through the original CBAM.

### 2.3. Loss Function

The loss function is one of the crucial elements of deep learning and is used for model training and evaluating. By measuring the difference between predicted and actual values through a loss function, the model can be adjusted to optimize parameters. In the context of semantic segmentation, cross-entropy is widely applied to this classification problem. To evaluate the difference between probability distributions of multi-categories, this study used the categorical cross-entropy (CCE) function, defined as follow:

(4) $$L_{CCE}\left(p,q\right)=-\frac{1}{N}\sum_{c=1}^{C}\sum_{i=1}^{n_{c}}p_{c,i}\log\left(q_{c,i}\right)$$

where *N* is the number of pixels, *C* is the number of categories, $n_{c}$ is the number of pixels in category C, p is the true label, and q is the predicted probability of the true class. In addition, the true labels in CCE function are one-hot encoded vectors, where the element corresponding to true class is 1 and all other elements are 0.

One of the most common problems encountered in deep learning tasks is the issue of dataset imbalance. In practical applications, datasets are rarely balanced, meaning that the amount of available data are seldom equal for all categories, such as medical images and SAR oil spill images. Due to the different proportions of data used for model training, the model is likely to be biased toward the majority class. As we provide a large amount of data for the majority class, the classification model will correctly learn to identify samples in the majority class. However, due to the insufficient data for the minority class, the model has poor predictive ability for such samples. As a result, the model tends to classify a large number of samples belonging to the minority class into the majority class. In other words, the model becomes overly confident in its predictions for the majority class samples. Label smoothing [36,37] reduces the model’s overconfidence in the majority class samples and also helps improve the model’s generalization ability. In this method, the labels of the training samples were modified to the following:

(5) $$y_{ls}=\left(1-\alpha \right)\times y_{l}+\alpha /K$$

where α is smoothing factor, $y_{ls}$ is the label after label smoothing, $y_{l}$ is original label, and *K* is number of categories. This study applied label smoothing to soften the true labels in the training data, preventing the deep learning model from overfitting and attempting to penalize overconfident outputs. This approach did not compromise the model’s performance while providing higher generalization ability. In the study, the α value was set to 0.1, because increasing the α value hindered the model’s ability to effectively converge during the training process, thereby reducing the detection performance.

### 2.4. Evaluation Metric

In the study, intersection over union (IoU) was used to evaluate the performance of the detection results, which is defined as follows:

(6) $$IoU=\frac{TP}{TP+FP+FN}=\frac{Groundtruth\cap Prediction}{Groundtruth\cup Prediction}$$

where TP and FP represent the number of correct and false pixels on the detection results, respectively. FN is the number of undetected pixels on the ground truth pixels. IoU is used to evaluate the performance of segmentation results by comparing the ground truth data with the predictions. In the experiments, IoU was measured for each category in the dataset, and mean IoU (mIoU) was computed as the average values of IoU across five categories.

## 3. Results

### 3.1. Experimental Setting

The following experiments were conducted using a PC with a 12th Gen Intel Core i7-12700KF CPU and 16 GB memory. In addition, the GeForce RTX3080 with 12 GB memory was employed, using CUDA v12.1 and cuDNN v8.8.0. The operating system was Windows 10 with a 64-bit processor. The deep learning model was built in the environment of TensorFlow-gpu version 2.10.1 and Keras version 2.10.0. In this study, the oil spill detection model was modified based on the U-Net model proposed by Chen et al. [23].

**Figure 5:**
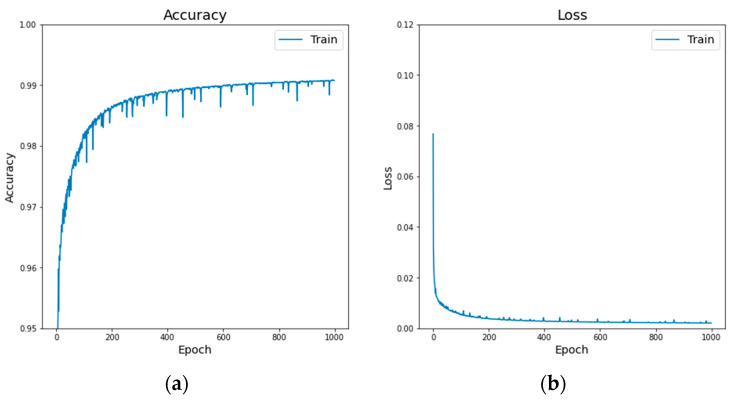
The training process of the improved FA-MobileUNet model. (**a**) Accuracy. (**b**) Loss.

The oil spill detection model was trained using the extended MKLab dataset [23], which consisted of 1239 images, with 1129 and 110 images in the training and testing sets, respectively. The original image size was 1250 × 650, which was resized to 352 × 352 as input for model training. During the training process, the number of epochs and batch size were set to 1000 and 8, respectively. In addition, the learning rate was set to 5 × 10^−5^. The Adam optimizer [38] and categorical cross-entropy were chosen as the optimization method and loss function, respectively. The training process of the improved FA-MobileUNet model was shown in Figure 5, reaching an accuracy of 0.9912 and a loss of 0.0008.

### 3.2. Performance Evaluation

The experiment first evaluated the performance of the modified CBAM in the improved FA-MobileUNet model for oil spill detection. The original CBAM modules of the FA-MobileUNet model were replaced by the modified CBAM from shallow to deep layers, and its performance was shown in Table 1. The FA-MobileUNet model using the original CBAM achieved an mIoU of 83.74%. By replacing the CBAM modules in stages 1 and 2 with the modified CBAM, the improved FA-MobileUNet achieved better detection performance, with an mIoU of 83.83%. However, if the CBAM in stage 3 was also replaced with the modified CBAM, the mIoU slightly decreased to 83.67%. Therefore, the study enhanced the performance of the improved FA-MobileUNet model by applying the modified CBAM to stages 1 and 2 of the encoding layers.

Since morphological operations, like convolution operations, can be iterated multiple times, it is necessary to select an appropriate number of iterations for the closing operation during model training. Next, the impact of iteration number of the closing operations in MAM on network performance was examined, as shown in Table 2. The FA-MobileUNet model using the original CBAM achieved 83.74% mIoU, with the IoU of oil spills and lookalikes reaching 75.85% and 72.67%, respectively. Then, different iterations (from 1 to 3 times) of the closing operation were performed in the modified CBAM of the improved FA-MobileUNet model. However, too many iterations of the closing operation may result in overestimation, negatively affecting the training of the model. It can be observed that performing two iterations of closing operations achieved better detection performance, with a slight improvement in the IoU for oil spills and lookalikes, reaching 83.94% mIoU.

Finally, label smoothing was applied to the improved FA-MobileUNet model, and its performance was verified. The detection performance of the improved FA-MobileUNet model with label smoothing was shown in Table 3. Although the improved FA-MobileUNet model achieved good detection results on different categories by enhancing multi-scale feature extraction, the use of label smoothing can still slightly improve the detection performance, especially for minority categories. Using label smoothing in the training process, the IoU of the oil spills and lookalikes increased by about 1.0% and 2.5%, respectively. Therefore, the experiments validated that label smoothing can improve the performance of the model and its generalization ability to imbalanced datasets.

### 3.3. Segmentation Network Comparison

The segmentation performance of the improved FA-MobileUNet model was compared with other semantic segmentation networks in this section. First, Krestenitis et al. [24] tested the MKLab dataset with different semantic segmentation networks, including U-Net, LinkNet, PSPNet, DeepLabv2, and DeepLabv3+. Similar to U-Net, LinkNet is a lightweight segmentation model that uses skip connections between the encoder and decoder. PSPNet employs a pyramid pooling module to capture multi-scale context by applying pooling operations at different grid sizes, aggregating both local and global information. DeepLabv2 introduces atrous (dilated) convolutions, which can expand the receptive field without losing resolution, and atrous spatial pyramid pooling (ASPP), which can achieve multi-scale feature extraction. DeepLabv3+, an enhanced version of DeepLabv3, incorporates an encoder–decoder structure, ASPP, and atrous convolutions for multi-scale context aggregation. Among them, except for DeepLabv3+ which used MobileNetv2 as the backbone network, the other networks used ResNet-101 as the backbone network. Fan et al. [14] proposed ToZero FMNet by combining a threshold segmentation algorithm and a feature merge network to segment oil spills. Basit et al. [39] proposed a new loss function about the gradient profile (GP) to train the model and combined it with the Jaccard and focal loss function, including CoAtNet-0 and EfficientNetv2 models. Rousso et al. [15] first applied different image filters to the training images, and then used the U-Net and DeepLabv3+ models for ensemble training to detect oil spills. Chen et al. [23] proposed the FA-MobileUNet model by incorporating CBAM, ASPP, and full-scale aggregated modules, and replacing the backbone architecture with MobileNetv3.

Table 4 summarized the performance evaluation of the models in terms of IoU. In [24], the U-Net model with a ResNet-101 backbone and a DeepLabv3+ with MobileNetv2 backbone achieved better detection results, with mIoU values of 67.40% and 67.41%, respectively. In the detection results of the U-Net and DeepLabv3+ models, the IoU of oil spills reached 57.07% and 56.34%, respectively. However, the DeepLabv2 model performed the worst in detecting oil spills, with an mIoU of only 56.37%. Therefore, models with an encoder–decoder architecture achieved better detection performance in this multi-class semantic segmentation task. Although ToZero FMNet incorporated different threshold segmentation methods into the U-Net model, its detection performance on the five categories in the MKLab dataset was worse than the original U-Net model. Next, CoAtNet-0 and EfficientNetv2 adopted a combination of GP, Jaccard, and focal loss functions during the training process, increasing the mIoU to 70%. However, the performance of these two models in detecting oil spills has not significantly improved, with IoU values of only 50.22% and 56.42%, respectively. Although the rnsemble model integrated the U-Net and DeepLabv3+ models for training, its detection performance for oil spills and lookalikes did not improve, with an mIoU of 71.12%. The FA-MobileUNet model enhanced multi-scale feature extraction, improving the IoU for oil spills, lookalikes, and ships to over 70%, reaching 83.67% mIoU. Finally, the improved FA-MobileUNet model with the modified CBAM and label smoothing method achieved the best detection performance, with an mIoU of 84.55%. The experiments validated the efficiency of the modified CBAM and label smoothing in detecting oil spills and lookalikes.

### 3.4. Oil Spill Detection Results Improvement

**Figure 6:**
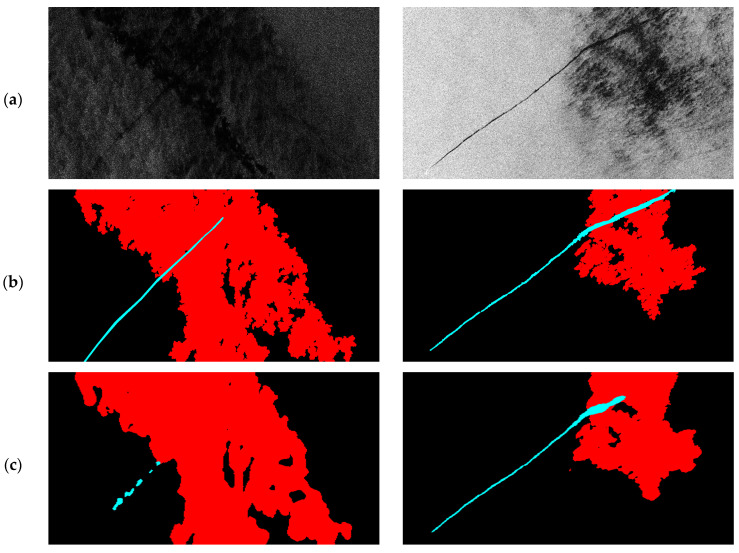

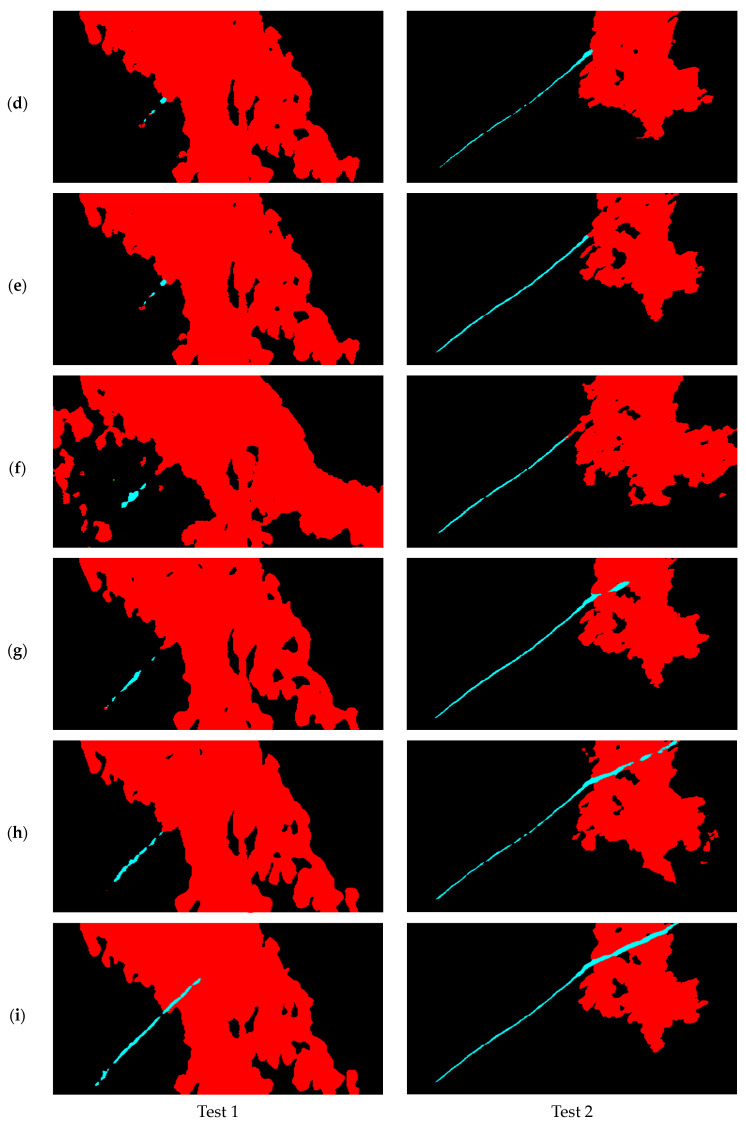
The segmentation results of different semantic segmentation models. (**a**) SAR image. (**b**) Ground truth data. (**c**) U-Net model. (**d**) LinkNet model. (**e**) PSPNet model. (**f**) DeepLabv2 model. (**g**) DeepLabv3+ model. (**h**) FA-MobileUNet model. (**i**) Improved FA-MobileUNet model. Black, cyan, and red represent the sea surface, oil spills, and lookalikes, respectively.

To further validate the performance of the improved FA-MobileUNet model and compare it with other semantic segmentation networks, the segmentation results of some test images were shown in Figure 6. The U-Net, LinkNet, PSPNet, DeepLabv2, DeepLabv3+, FA-MobileUNet, and the improved FA-MobileUNet models were used to detect the oil spills in test images. Black, cyan, and red represent the sea surface, oil spills, and lookalikes, respectively. As shown in Figure 6a, the black areas of oil spills overlapped with lookalikes in the SAR images, making identification difficult for these semantic segmentation models. In addition, the original ground truth data in Figure 6b showed that the oil spills and lookalike areas were somewhat fragmented. From the detection results of the Test 1 image, the improved FA-MobileUNet model can detect the oil spill area more accurately and completely, compared to the fragmented detection results of other models. LinkNet, PSPNet, DeepLabv2, and DeepLabv3+ cannot effectively detect oil spill areas. While the U-Net model detected part of oil areas, it also found it difficult to accurately distinguish oil spills from lookalikes. Moreover, compared to the FA-MobileUNet, the improved FA-MobileUNet model provided more complete detection results for the lookalike areas, with some smaller holes filled. Observing the detection results of Test 2 image, although all these models detected oil spills, only the U-Net, DeepLabv3+, FA-MobileUNet, and improved FA-MobileUNet models identified some overlapping areas. Among them, the improved FA-MobileUNet model detected the most complete oil spill area. Although the modified CBAM seemed to have limited improvement in mIoU from the previous experimental results of Section 3.2, it was observed that FA-MobileUNet combined with the modified CBAM can achieve more complete oil spill detection, with fragmented hole areas being filled. The experimental results validated that the improved FA-MobileUNet model can effectively detect oil pollution even in complex scenes. Therefore, this model can be applied to practical oil pollution incidents to verify its effectiveness in oil pollution identification.

### 3.5. Oil Pollution Incidents

**Figure 7:**
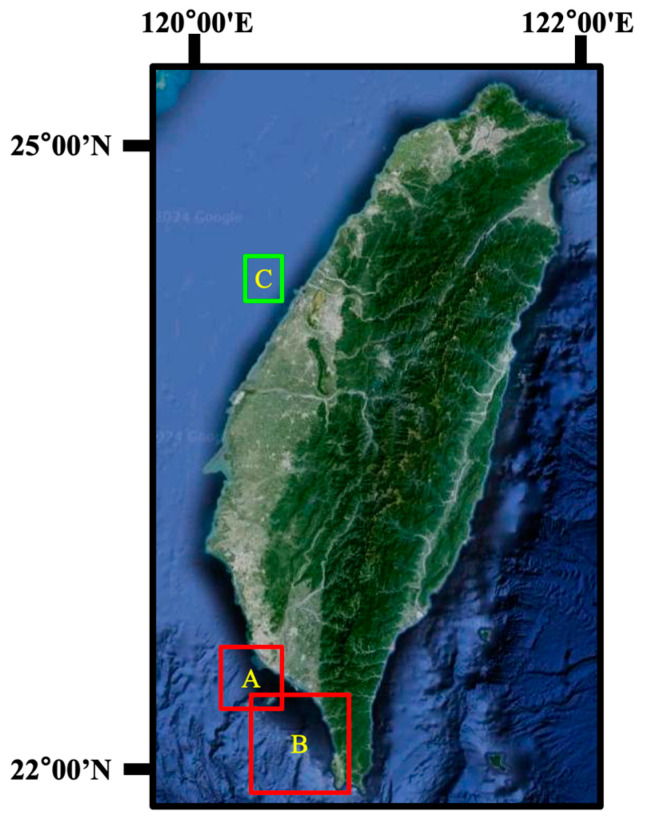
The areas of oil pollution incidents in Taiwan. Regions A–C are located near the Kaohsuing Port, Xiaoliuqiu Island and Taichung Port, respectively.

The experiments in this section were conducted to verify the effectiveness of the proposed oil spill detection model through oil pollution incidents. In recent years, two serious oil pollution incidents have occurred in Taiwan’s marine areas. One pollution incident, caused by a shipwreck, occurred in Region A of Figure 7, close to the Kaohsiung Port. The other pollution incident, caused by the rupture of an undersea oil pipeline, occurred in Region B of Figure 7, near Xiaoliuqiu Island. Kaohsiung Port is the international port with the largest container throughput in Taiwan. Therefore, most oil pollution incidents in this port were caused by ships. Moreover, there is an oil refinery along the coast of Kaohsiung. When an underwater oil pipeline rupture occurred, the oil spills spread to the nearby coastline of Xiaoliuqiu Island and Kenting. Both of the abovementioned incidents seriously damaged Taiwan’s marine environment and affected the survival of marine life, and a large amount of manpower and resources were spent on oil pollution cleanup. If oil pollution incidents can be properly monitored, damage to the marine environment and marine life can be mitigated.

#### 3.5.1. Oil Pollution Caused by Shipwreck

**Figure 8:**
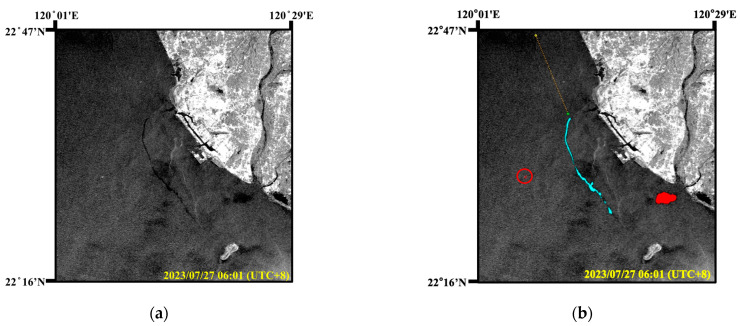
Oil spill detection results caused by a shipwreck. (**a**) SAR image; (**b**) oil spill detection results (oil spills: cyan, lookalikes: red, and ships: brown).

First, an oil pollution incident, caused by a shipwreck, occurred in Region A of Figure 7. On 21 July 2023, a Palau-flagged container ship named ANGEL sank due to water ingress into the cabin, causing the hull to tilt. According to the incident report, the ship carried a total of 491.848 metric tons of oil. Therefore, the shipwreck resulted in oil pollution in the surrounding marine areas, as shown in the black area in Figure 8a. The Sentinel-1 image was captured at 06:01 on 27 July 2023 (UTC + 8), with an image size of 1320 × 1409.

On 24 June 2023, the container ship ANGEL departed from Dalian Port in China after loading containers. However, before departure, the ship had already experienced water ingress due to a hull breach and the ballast water system was temporarily used to pump out the water. During its voyage, the ship sent a message to the South Taiwan Maritime Affairs Center of the Maritime Port Bureau (MPB), requesting permission to enter Kaohsiung Port for inspection and repairs. On 2 July, the ship arrived and anchored offshore of Kaohsiung Port, as indicated by the green circle in Figure 8b. However, MPB rejected the request for repairs, so the container ship ANGEL remained anchored in place. On 20 July, a generator on ANGEL malfunctioned, causing a loss of power. Consequently, the ballast water system failed to operate and the accumulated water could not be pumped out, causing the ship’s hull to gradually tilt until it completely sank on the morning of 21 July.

The acquired Sentinel-1 image was in EW mode with a resolution of 40 m × 40 m. Oil spills, lookalikes, and a ship (marked by a red circle) were detected in the SAR image by the proposed model. The oil spill detection results covered a total of 3621 pixels, which was approximately 5.79 square kilometers. It was not until Typhoon DOKSURI passed through on 29 July that maritime monitoring was conducted, revealing the oil spill area to be at least about 1.95 square kilometers. Therefore, the use of SAR images and the oil spill detection model can effectively monitor the marine environment without being affected by weather conditions.

#### 3.5.2. Undersea Oil Pipeline Rupture Incident

**Figure 9:**
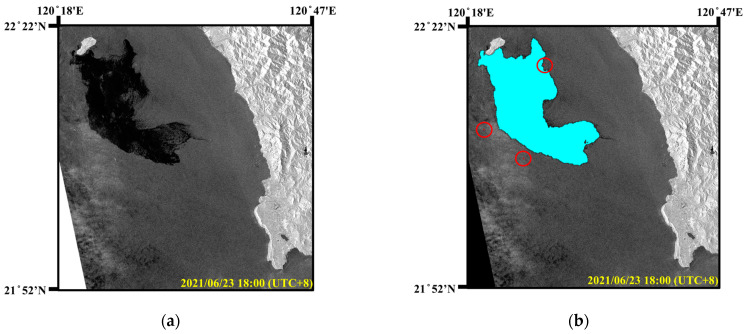
Oil spill detection results in Xiaoliuqiu Island. (**a**) SAR image. (**b**) Oil spill detection results (oil spills: cyan, and ships: brown).

There is a Dalin oil refinery along the coast of Xiaogang, Kaohsiung. In the early morning of 22 June 2021, an offshore oil pipeline ruptured. Due to the rain and poor visibility, it was impossible to visually identify the oil spills on the sea surface. As the ocean current in this area flows to the southeast, the oil spills drifted towards Xiaoliuqiu. As shown in Figure 9a, the Sentinel-1 image on 23 June 2021 clearly showed the oil spills near the marine area of Xiaoliuqiu island located in Region B in Figure 7. The acquired Sentinel-1 image was in IW mode with a resolution of 10 m × 10 m and an image size of 5369 × 5555. According to records of the oil spill incident, the oil spills covered an area of approximately 290 km^2^. As shown in Figure 9b, the detection results in the SAR image included ships (in red circles) and oil spills. The detected oil spill area was 2,889,041 pixels, approximately 288.9 km^2^.

**Figure 10:**
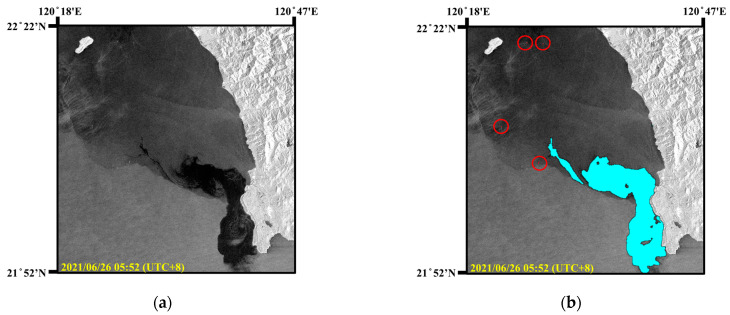
Oil spill detection results in Kenting National Park. (**a**) SAR image. (**b**) Oil spill detection results (oil spills: cyan, and ships: brown).

Subsequently, the oil spills continued to drift southeastward and spread to the coast of Kenting National Park, severely affecting the local ecosystem and fishery resources. As shown in Figure 10a, the Sentinel-1 image on 26 June 2021, revealed the oil spill area. According to the oil spill detection results in Figure 10b, this area contained 1,829,760 pixels, approximately 182.9 km^2^. Consequently, this pollution required extensive manpower for cleanup and caused serious environmental damage to coastal areas. In summary, the proposed oil spill detection model based on SAR images can realize all-day and all-weather marine environment monitoring and provide real-time oil spill information.

## 4. Discussion

### 4.1. Evaluation of the Morphological Attention Module

Although the FA-MobileUNet model [23] incorporated the spatial pyramid model and attention mechanism can effectively improve the accuracy of oil spills and lookalikes detection, there were still some fragments and small holes present in the detection results. In order to facilitate the description of the spread range and area measurement of oil spills when an oil pollution incident occurs, it is necessary to detect a relatively complete oil pollution area. However, post-processing of the detection results might cause smaller ocean targets, such as ships, to disappear. To address this issue, the improved FA-MobileUNet model incorporated morphological concepts into CBAM, achieving more complete detection areas. Therefore, experiments were conducted on the FA-MobileUNet model and the improved FA-MobileUNet model combined with the modified CBAM for explicit comparison. Additionally, the experiments also compared the impact of different iterations of the closing operation in the modified CBAM on the detection results.

**Figure 11:**
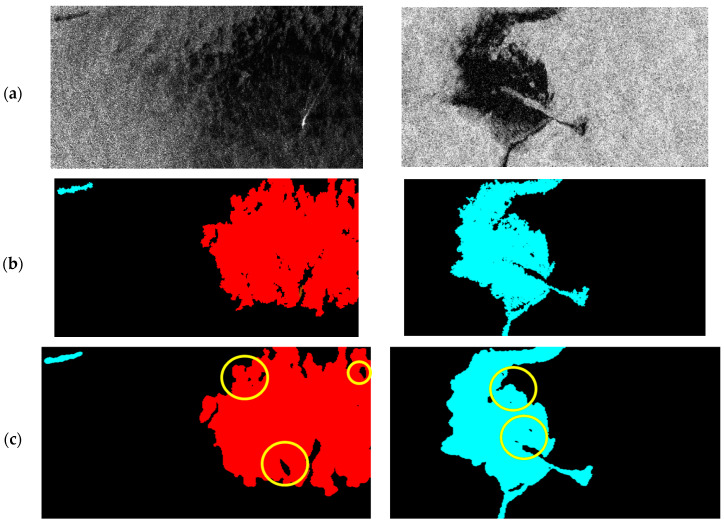

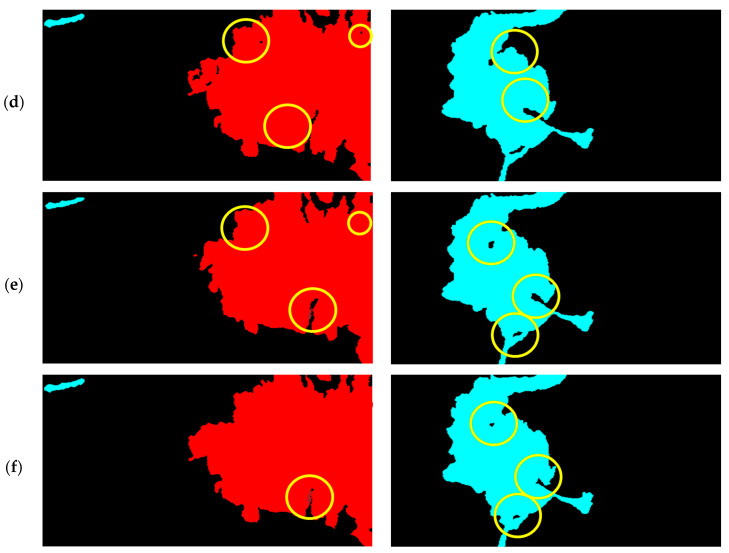
Comparison of the segmentation results by the proposed model combined with the original and modified CBAM, respectively. (**a**) SAR image; (**b**) ground truth data; (**c**) the FA-MobileUNet model using original CBAM; (**d**) the improved FA-MobileUNet model using modified CBAM (with 1 iteration of the closing operation). (**e**) The improved FA-MobileUNet model using modified CBAM (with 2 iterations of the closing operation). (**f**) The improved FA-MobileUNet model using modified CBAM (with 3 iterations of the closing operation). Black, cyan, red, and brown represent the sea surface, oil spills, lookalikes, and ships, respectively.

The segmentation results of the improved FA-MobileUNet model combined with the modified CBAM and the FA-MobileUNet were shown in Figure 11. It can be observed that the labeled data were fragmented due to severe sea clutter, as shown in Figure 11b, because they have not been post-processed. There were still some small holes in the detection results of FA-MobileUNet model, which was combined with the original CBAM, as shown in Figure 11c. The study referred to the concept of morphology to modify the spatial attention module in the CBAM. Most of the small holes in the detection results can be filled through the improved FA-MobileUNet model combined with the modified CBAM, in which the closing operation was performed once, as shown in yellow circles in Figure 11d. However, there were still some unfilled holes in the detection results. Next, the improved FA-MobileUNet model improved the hole filling in the detection results and reduced the size of larger holes by iterating the closing operation twice, as shown by the yellow circles in Figure 11e. However, the improved FA-MobileUNet model using three iterations of closing operations overfilled the holes, resulting in greater discrepancies compared with the ground truth data, as shown in Figure 11f. Therefore, overfilling the holes will result in the loss of some object details, affecting object detection performance. The experimental results showed that the CBAM incorporating the concept of morphology into the improved FA-MobileUNet model can obtain more complete detection results, but excessive closing operations may affect the overall performance. Therefore, the improved FA-MobileUNet model, using the modified CBAM with two iterations of the closing operation, was able to appropriately fill the holes and achieve better detection results.

### 4.2. Predicting Suspicious Oil-Discharge Ship Combining SAR and AIS

Oil pollution from ship accidents or illegal discharges occurs more frequently in the marine environment [2]. SAR images can be used to observe oil pollution incidents, but they cannot provide detailed information about the ships suspected of leaking oil. Through the proposed oil spill detection model, extensive observations and monitoring of the marine environment can be fulfilled. However, to effectively discover clues of oil spills that may be discharged by ships, it is not only necessary to improve oil spill detection performance but also to require detailed navigation information of ships. The automatic identification system (AIS) is an automatic tracking system installed on ships that regularly transmits ship navigation-related information, including static information, such as vessel name, length, and width, as well as dynamic information, such as position (latitude and longitude), speed, heading, and course. However, AIS data are only available on vessels equipped with AIS receivers, and AIS signal overlap and loss may occur in areas with dense concentrations of ships. While individual technologies may have inherent limitations, the combination of SAR and AIS data can provide more information for effective monitoring of vessel activities. The studies in [40,41] have shown that the combination of SAR and AIS data can effectively monitor the marine environment and track vessels suspected of discharging oil spills.

**Figure 12:**
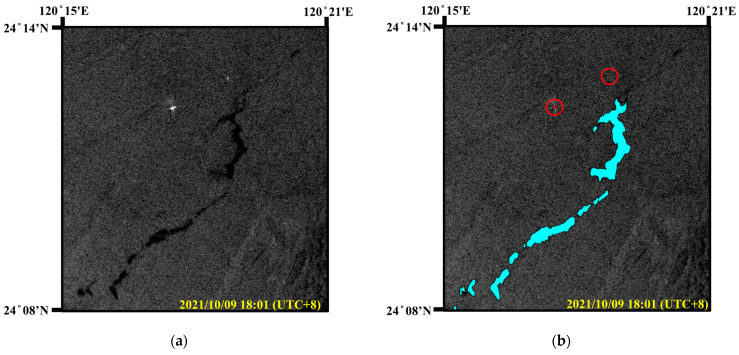

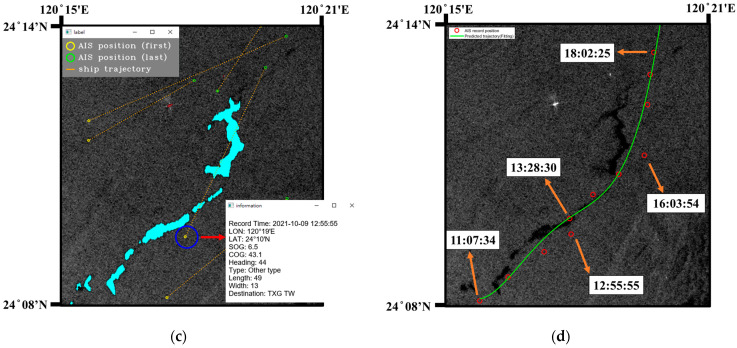
Tracking ships suspected of discharging oil spills using SAR and AIS data. (**a**) SAR image. (**b**) Oil spill detection results (oil spills: cyan, and ships: brown). (**c**) Ship trajectories provided by AIS (in orange dotted line). (**d**) Trajectory of suspected oil-discharging ship.

During the collection of SAR oil spill images, it was found that one SAR image included the oil spill area near Taichung Port, Taiwan, as shown in Region C in Figure 7. The Sentinel-1 IW mode SAR image containing the oil spills was taken at 18:01 on 9 October 2021 (UTC + 8), with an image size of 1171 × 1252, as shown in Figure 12a. In fact, the oil pollution area is still a certain distance from Taichung Port, and there are no submarine oil pipelines nearby. Therefore, the oil spills caused by port operations or pipeline leaks are less likely happened. Moreover, Taichung Port is Taiwan’s second largest international port in terms of container throughput, and many container ships and oil tankers often navigate and anchor in the area. Some shipping companies or captains sometimes discharge waste oil directly into the ocean before entering the port in order to save the operating costs of pollution prevention and control equipment. Through the following analysis, this study combined SAR and AIS data to find the possible cause of the oil spill.

Initially, the proposed improved FA-MobileUNet model was used to detect targets in this SAR image and successfully identified the oil spill areas and the ships (red circled area), as shown in Figure 12b. Since the ship was far from the oil spill area, it could not be confirmed whether this ship discharged the oil spills. Next, in order to check whether the oil spill was caused by the ship, the study collected the AIS data corresponding to this area based on the timestamp of the Sentinel-1 image. AIS records were collected within 7 h before the image acquisition time to obtain the ship trajectories. The superposition result of the detected oil spills and AIS data were shown in Figure 12c. This study filtered the position of each ship from the AIS data through their MMSI (maritime mobile service identity) and marked the first and last reported AIS positions during the period with yellow and green circles, respectively. These two points were connected by an orange dot line, which represents the approximate trajectory of the ship. From the results in Figure 12c, it can be observed that the ship marked by the blue circle was closest to the oil spill, and its trajectory intersected with the oil-covered area. Then, a more accurate navigation trajectory of this ship was estimated through more AIS information, including long-term position, speed, heading angle, etc. In Figure 12d, the estimated ship trajectory was fitted to a quartic curve and shown as the green line, and the long-term positions of the suspicious vessel reported by AIS were shown by red circles. From the results in Figure 12d, it can be found that the ship’s navigation trajectory was quite consistent with the oil spill spreading direction. According to the experimental results, the oil spill was probably caused by the suspicious ship. The above analysis showed the potential of combining SAR and AIS data to find possible causes of oil spills. Clues about suspected oil discharge vessels can be provided to the Coast Guard to increase the alertness of shipping companies or crews and reduce illegal oil discharge pollution incidents. Therefore, the proposed oil spill detection approach can effectively enhance the protection of the marine environment.

## 5. Conclusions

This study proposed an improved FA-MobileUNet model for maritime oil pollution monitoring. The proposed model modified the original CBAM in the FA-MobileUNet model into a morphology-based attention module. Experimental results showed that the modified CBAM can obtain a more complete oil spill detection area, which helps describe the spreading range of oil spills when an oil pollution incident occurs. The results also showed that the modified CBAM, which underwent two iterations of morphological closing operations and was applied to the first and second layers of the U-Net encoder, performed better in extracting target structures during training. Additionally, the proposed model further improved the detection performance by performing label smoothing during training, improving the IoU of oil spills and lookalikes by approximately 1.0% and 2.5%, respectively. Compared with other semantic segmentation networks, the improved FA-MobileUNet model achieved efficient oil spill detection performance, reaching an mIoU of 84.55%. In particular, the IoU corresponding to oil spills and lookalikes were 1.65% and 3.12% higher than those of FA-MobileUNet, respectively. Experimental results also showed that the proposed model can obtain a more complete detection area of oil spills and lookalikes by filling holes and eliminating fragmented regions. Moreover, the effectiveness of the improved FA-MobileUNet model in oil spill detection was further verified through two serious oil pollution incidents which occurred in the western marine region of Taiwan. The oil spill detection results closely matched the oil pollution areas recorded in the incidents, verifying that the improved FA-MobileUNet model can effectively monitor marine oil pollution. Finally, it was discussed that the oil spill detection model combined with SAR and AIS data has the potential to find clues about suspicious oil-discharge vessels, thereby strengthening the supervision of the marine environment. The effectiveness of the proposed oil pollution monitoring method combining SAR and AIS data is worth exploring and will be investigated in future studies.

## Figures and Tables

**Table 1:** Detection performance of the improved FA-MobileUNet model with different replacement of the modified CBAM in terms of IoU (%). “
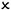
” represents the model using original CBAM.

Modified CBAM		Sea Surface	Oil Spills	Lookalikes	Ships	Land	mIoU
Encoder stage	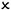	97.54	75.85	72.67	76.19	96.48	83.74
	1	97.31	75.86	72.91	76.19	96.49	83.75
	1, 2	97.22	75.97	73.25	76.21	96.49	83.83
	1, 2, 3	96.03	76.34	73.39	76.20	96.40	83.67

**Table 2:** Detection performance of the improved FA-MobileUNet model under different closing operation iterations in the modified CBAM in terms of IoU (%). “
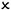
” represents the model using original CBAM.

Module	Iteration No.	Sea Surface	Oil Spills	Lookalikes	Ships	Land	mIoU
Modified CBAM (closing operation)	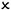	97.54	75.85	72.67	76.19	96.48	83.74
	1	97.32	75.94	73.52	76.22	96.47	83.89
	2	97.08	76.12	73.88	76.22	96.40	83.94
	3	94.85	76.88	74.13	76.23	96.38	83.69

**Table 3:** Performance evaluation of the improved FA-MobileUNet using label smoothing in terms of IoU (%).

Method		Sea Surface	Oil Spills	Lookalikes	Ships	Land	mIoU
Label smoothing	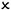	97.54	75.85	72.67	76.19	96.48	83.74
	✓	97.29	76.84	75.21	76.42	96.45	84.44

**Table 4:** Performance evaluation of different semantic segmentation networks in terms of IoU (%). Bold fonts represent the best detection results for the category.

Model	Backbone	Parameters	Sea Surface	Oil Spills	Lookalikes	Ships	Land	mIoU
U-Net	ResNet-101	51.5 M	95.47	57.01	44.82	46.62	93.08	67.40
LinkNet	ResNet-101	47.7 M	94.82	52.95	47.52	45.11	93.12	66.70
PSPNet	ResNet-101	3.8 M	93.03	45.65	40.62	30.25	91.12	60.13
DeepLabv2	ResNet-101	42.8 M	95.02	43.12	46.23	15.12	82.34	56.37
DeepLabv3+	MobileNetv2	2.1 M	96.57	56.34	57.06	32.92	94.18	67.41
ToZero FMNet	x	36.0 M	94.53	49.95	41.40	25.44	87.11	61.90
CoAtNet-0	x	29.4 M	95.40	50.22	58.85	69.09	94.49	73.61
EfficientNetv2	B1	16.7 M	95.19	56.42	62.23	72.80	**96.59**	76.65
Ensemble Model	x	x	96.78	56.10	58.88	47.28	**96.59**	71.12
FA-MobileUNet	MobileNetv3	14.9M	**97.12**	75.85	72.69	76.22	96.47	83.67
Improved FA-MobileUNet	MobileNetv3	14.9M	96.58	**77.50**	**75.81**	**76.67**	96.18	**84.55**

## Data Availability

The data presented in this study are available upon request from the author. The data are not publicly available due to privacy restrictions.
